# Aging behavior of polymeric-fiber based vacuum insulation panels

**DOI:** 10.1039/d5ra02871j

**Published:** 2025-08-21

**Authors:** Jianzhu Ju, Changxi Li, Qunjian Huang

**Affiliations:** a Hefei Hualing Co., Ltd, Midea Group Hefei 340100 China jianzhu.ju@outlook.com licx198@midea.com

## Abstract

For vacuum insulation panels (VIPs), aging resistance decides the long-term reliability of the products, which is critical for appliance and building applications. As solid and gaseous conduction in VIP has opposite dependence on core material porosity, it is challenging to achieve desired performance in both initial and persistent insulation. In this work, a novel VIP with ultrathin polymeric fiber (diameter of 7 μm) as core material is developed, in which low initial thermal conductivity down to 1.6 mW mK^−1^ can be achieved with moderate porosity around 85%. Characterized by *in situ* thermal conductivity and pressure measurements, polymeric fiber-based VIP features a two-phase aging behavior: the short-term aging is governed by the viscoelasticity of the polymeric fiber, and partially reversible upon heating; the long-term aging is dominated by gas permeation, with a low rate around 0.1 mW per mK per year at room temperature. With the excellent combination of low initial conductivity and strong aging resistance, polymer fiber-based VIPs provide a cleaner alternative to traditional VIP core materials, with further possibility to be explored.

## Abstract 2

Polymeric fiber VIP achieves ultralow initial thermal conductivity and slow aging. *In situ* pressure and thermal conductivity tests reveal the aging mechanism of VIP. Aging of polymeric fiber VIP depends on both viscoelasticity and gas permeation.

## Graphical abstract

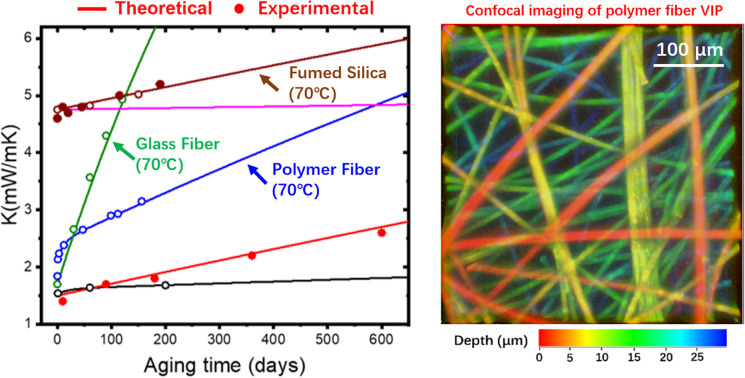

## 1. Introduction

Vacuum insulation panels (VIPs) are a novel insulation material that can achieve ultralow thermal conductivity *K* below 2 mW mK^−1^.^1–5^ The advantage of VIPs over other insulation material comes from its evacuated microscopic pores, where Knudsen effect^6–8^ largely suppresses the gas conduction and high porosity decreases the solid conduction. As a novel insulation material that has been increasingly applied by major appliance manufacturers,^9–13^ the biggest concern about the practical application of VIPs is the long-term reliability.^1,2,4,14–18^

The performance degradation of VIPs (increasing *K* over service time) has been attributed to the increasing gas conduction from gas/water permeation^19–21^ and core material outgassing.^22–24^ The dependence of gaseous conductivity *K*_g_ on pressure can be approximately described by^25–27^1
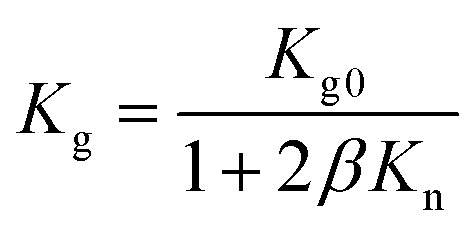
*K*_g0_ is gaseous conductivity in atmosphere, *β* is the dimensionless coefficient (in the order of 1) describing the collision between the molecular molecule and the pore.^25,27^ Knudsen number *K*_n_^6,25,28,29^ (the ratio of mean free length *l* and the size of structural boundary *δ*) characterizes the interaction between the gas molecule and the rigid boundary:2
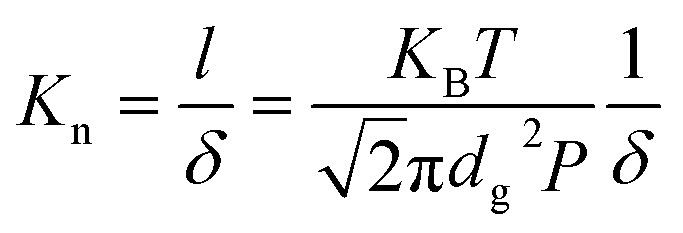
, where *K*_B_ = 1.38 × 10^−23^ J K^−1^ is Boltzmann constant, and *d*_g_ is the diameter of the gaseous molecule. *l* describes the mean distance that molecule travels between two collisions at temperature *T* and pressure *P*. During aging, the increasing *K*_g_ is essentially due to the additional gas that decreases *K*_n_ and benefits the heat transfer between gas molecules (intermolecular collision).

The resistance to aging depends on the core material structure of VIPs. From eqn (1), with increasing *l* under low *P*, *K*_g_ increases linearly with *P* (*K*_n_ ≫ 1 in VIP condition). Importantly, a core material with smaller *δ* shows better aging resistance, *i.e.*, smaller Δ*K*_g_/Δ*P*. On the other hand, with same size of the base element *d*_be_, solid conduction grows with decreasing *δ* (larger porosity 
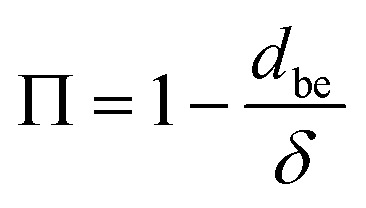
).^30^ This indicates that the initial and persistent insulation have opposite dependence on VIP porosity from the same base element. The aging behavior of two types of the most common core materials (glass fiber (GF) and porous silica (*e.g.* fumed silica and aerogel)) of VIP are very different. For GF, ultralow conductivity down to 1.25 mW mK^−1^ can be achieved with high porosity around 90%.^3,31^ Unfortunately, *K* aging is rather rapid, in the range of 0.3–0.5 mW per mK per year.^31,32^ As for porous silica VIP,^33–35^ fumed silica or aerogel based VIP possesses *K* around 4 mW mK^−1^,^33,36^ much higher than that of GF based VIP. However, porous silica based VIP shows excellent aging resistance with *K* increasing rate of 0.01–0.1 mW per mK per year.^21,37^ In this way, the performance of porous silica-based VIP remains almost unchanged in 10 years and drops only 40% in 30 years. The superior aging performance of porous silica based VIP is due to their smaller close-cell pore size (2 to 50 nm,^38^ which is unevacuatable). Practically, the aging resistance is somehow superfluous, especially for appliance industry. The trade-off between initial *K* and aging resistance needs to be addressed for the future application of VIP.

In this work, a novel core material for VIP production composed of polymeric fiber (PF) is developed and compared with GF and glass fiber-fumed silica composite. A two-phase aging behavior is observed in PF based VIP. Combining creep test of PF and *in situ* pressure measurements of different VIP, the short-term and long-term aging can be well described and predicted by an analytic model based on viscoelasticity, pore size and gas permeation rate. PF based VIP with an intermedia pore size shows superior aging resistance over GF, while possessing comparable initial *K* (ranges from 1.5 to 2.0 mW mK^−1^). Aging of 0.1 mW per mK per year can be obtained, so that 2.5–3 mW mK^−1^ after 10 years should be maintained (60%∼66% of remaining insulation capability). The ideal combination of initial *K* and aging resistance shows the advantageous of PF over previously reported core materials.

## 2. Materials and methods

### 2.1 Manufacturing process of VIP

**Fig. 1:**
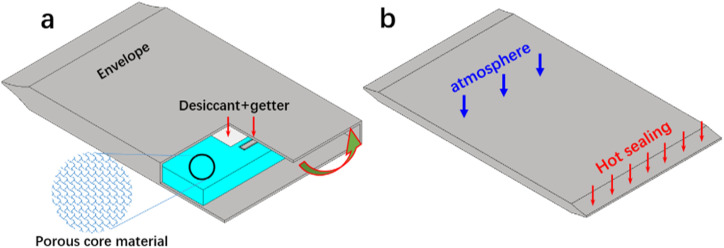
Schematic drawing of the encapsulation of VIP: (a) before sealing, porous core material is wrapped into barrier envelope, with desiccant and getter inserted; (b) encapsulated VIP after sealing, in atmosphere pressure.

The evacuation and sealing process of VIP is shown in Fig. 1. The porous core materials (desiccant and getter are inserted (Fig. 1(a)) are cut into 34 cm × 44 cm with different original thickness (calculated based on 1 cm thickness after compression in atmosphere). Then the core material is wrapped into the gas barrier envelope and evacuated in the vacuum chamber. The barrier envelope typically contains polyethylene terephthalate (PET) as substrate, polyurethane (PU) as glue, ethylene vinyl alcohol (EVOH) and aluminum (Al) as barrier layer.^39,40^ In our work, the two surfaces of the VIP are covered by different barrier layers: one side contains Al with thickness of 7 μm, and the other side contains EVOH with thickness of 12 μm, and deposited by Al layer of 38 nm. The designing purpose of the different layers is to decrease the effect of thermal bridge.^41,42^ The chamber is equipped with roots pump and diffusion pump, with capability of low pressure down to 10^−3^ Pa around 30 minutes. After reaching desired chamber pressure, the gas barrier envelope is sealed by the heat pressing of the PE layer. Back in atmosphere, the sealed VIP is compressed and obtained structural strength (Fig. 1(b)), making it suitable for application purpose. The whole encapsulation process is performed under room temperature and humidity.

### 2.2 Core material characterization

Three different core materials of VIP are applied in this work: (1) polymeric fiber (PF), (2) glass fiber (GF) and (3) glass fiber-fumed silica composite (GF-FS). PF used in this work are composite fibers is commercialized product in Midea group. PF are thoroughly dispersed first to produce the highly porous core material with porosity of 83%. The density of GF is 2501 kg m^−3^, measured by the volume increase after dispersing the GF in water and sonicating for 20 minutes. The porosity of GF is calculated to be 88%. GF-FS core material are commercially available product. For GF-FS, the fumed silica (density below 50 kg m^−3^, compared to 2200–2600 kg m^−3^ for silicon dioxide) has weight concentration of 40%, so that majority of volume concentration.

**Fig. 2:**
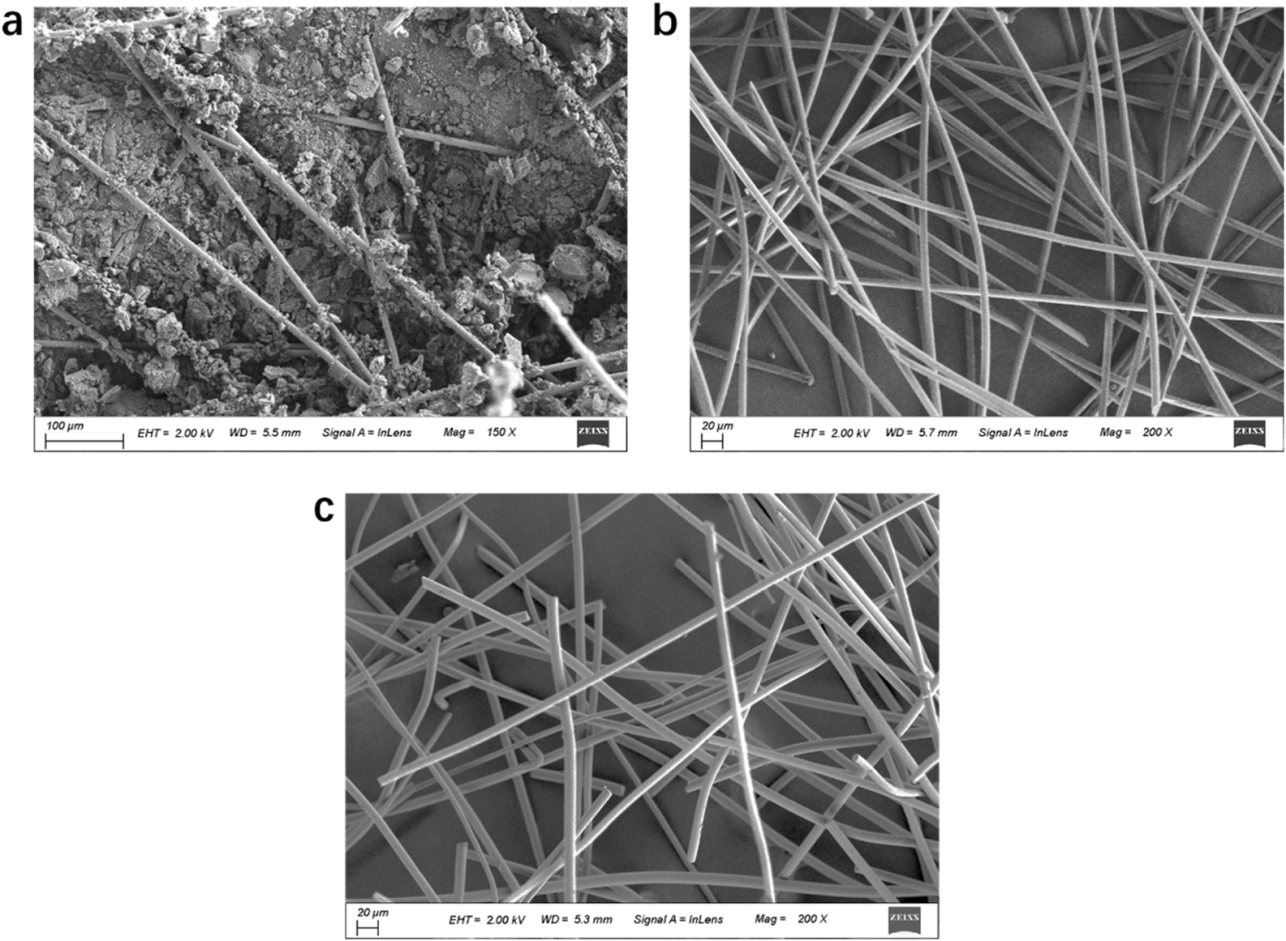
SEM images of one slice of core material: (a) GF-FS, (b) PF and (c) GF.

The scanning electronic microscopy (SEM) images of the sliced core material by GF-FS, GF and PF are shown in Fig. 2(a)–(c), respectively. SEM images are collected on Gemini SEM 500.^43^ In Fig. 2(a), aggregation of fumed silica can be observed with GFs in between. The majority of the core material is composed of fumed silica and the pore size is much smaller than that in fibers (typically in the range of 2 to 50 nm (ref. 38)). The diameter of PF and GF can be calculated from Fig. 2(b) and (c), around 6.8 μm and 9.4 μm, respectively.

### 2.3 Confocal microscopy of evacuated core material

Core material for confocal microscopy measurements are first dyed by 10 drops (around 0.2 mL per drop) of fluorescent suspensions at the same location for observation. Fluorescent suspension used in this work is polystyrene nanoparticles dispersed in water (diameter 100 nm, concentration 10 mg mL^−1^) with excitation and emission wavelength of 488 nm and 518 nm, respectively. The core material is fully dried under 50 °C and encapsulated in transparent PE envelope (thickness 10 μm). Conformal microscopy is measured right after encapsulation to avoid gas permeation. The 2D images of the core material are collected at different depth from the surface to the deepest detectable layer. The 2D images are coded in different color based on depth to demonstrate the 3D distribution of fibers in the evacuated core material.

### 2.4 Thermal conductivity measurement

The thermal conductivity (*K*) is measured by JW-III, produced by Beijing Jianyan Tianrun Technology Co., Ltd VIPs with thickness of 1 cm are hold between the cold (5 °C) and hot plate (35 °C) for over 2 hours and *K* value is normally stabilized after 1 hour. *K* is calculated based on:^44,45^3
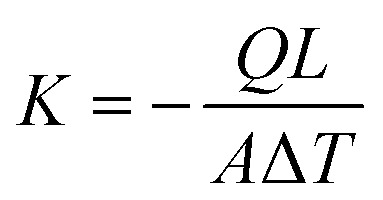
where Δ*T* is the temperature thickness, *Q* is the heat flux, *A* is the cross-section area and *L* is the thickness.

### 2.5 Gas pressure measurement

**Fig. 3:**
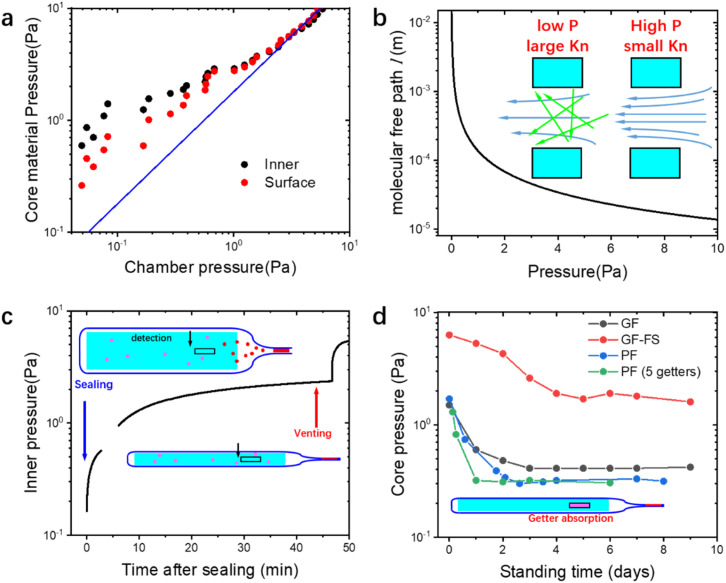
(a) Inner pressure and surface pressure of the core material, as the function of chamber pressure, respectively. (b) Molecular free path *l* for pressure below 10 Pa. Inset: schematic of Knudsen effect (gas flow under low and high pressure). (c) Inner pressure of the core material after sealing and venting of the chamber. Inset: schematic of gas molecule distribution after sealing and venting. (d) Core pressure (surface of the core material) as the function of standing time after penetrating the getter path. Inset: schematic of gas molecule distribution after activation of the desiccant.

**Fig. 4:**
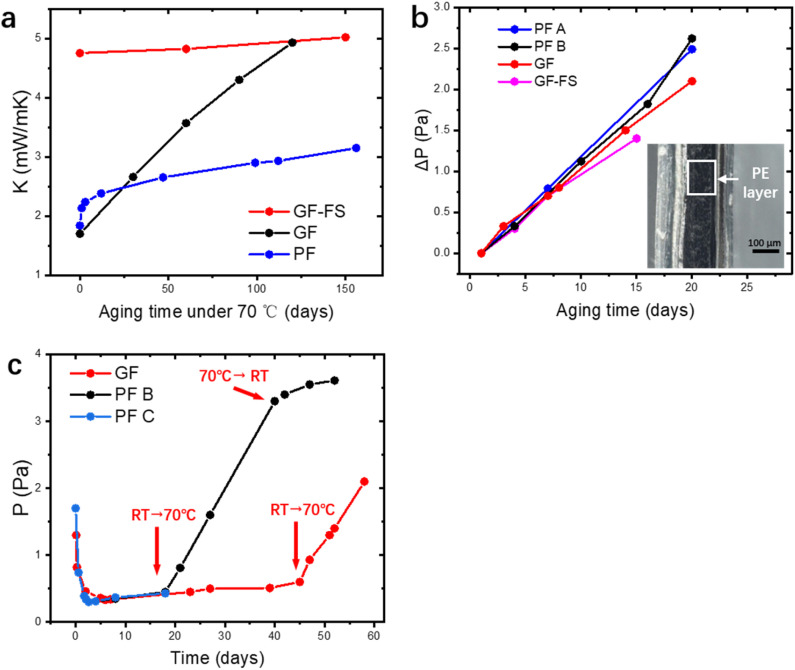
(a) Thermal conductivity as the functions of aging time at 70 °C. (b) Extracted pressure increase as the function of ageing time at 70 °C. Inset: Microscopy image of the cross-section of the sealed envelope, with polyethylene layer indicated. (c) Pressure increase of three different fibers during room temperature process and 70 °C process.

**Fig. 5:**
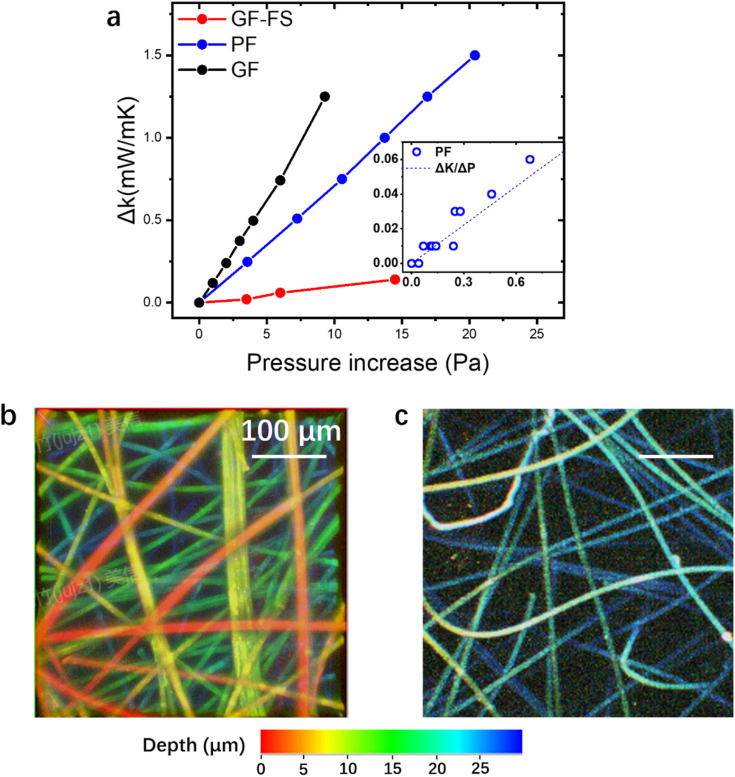
(a) Δ*K* as the function of Δ*P* at room temperature. Inset: linear relationship of PF in the small Δ*P* range. (b) Depth-coded image of PF based VIP in vacuum condition. (c) Depth-coded 3D image of GF based VIP in vacuum condition.

For gas pressure measurements with time resolution requirement (Fig. 3 and 5), PRG500 Pirani gauge developed by Infitech is applied to provide high time resolution and wide pressure detection range (0.05–10^5^ Pa). In Fig. 4, the inner pressure of the VIP is measured by ZJ-10 ionization gauge (detection range: 3 × 10^−4^–65 Pa) by Chengdu Zhenghua Electronic Instrument Co., which is applicable up to 80 °C.

### 2.6 Dynamic mechanical analysis

Dynamic mechanical analysis (DMA) results are tested on a DMA 242 E Artemis by Netzsch. Creep tests are performed with applied stress of 0.15 GPa and one single bundle of fibers is held for 30 minutes after reaching desired temperature.

## 3. Pressure involvement during and after the VIP encapsulation

The typical evacuation in VIP manufacturing requires an evacuation time below 30 minutes and final chamber pressure below 10^−2^ Pa. It is natural to assume the pressure inside VIP to be same as the chamber pressure, while this is only valid at relative high pressure. The flow of gas molecule is at low pressure highly limited under Knudsen effect,^6^ and the evacuation at low pressure is extremely difficult. To examine the evacuation efficiency, the inner pressure and the surface pressure of the PF core material are measured during evacuation with the reference of the chamber pressure, as shown in Fig. 3(a). It can be discovered that for pressure over 2 Pa, the inner and surface of the core material have same pressure, but smaller than the chamber pressure. For pressure below 2 Pa, the pressure drop of the core material (surface and inner) is significantly slower than the chamber. At chamber pressure of 0.04 Pa, the inner and surface pressure are around 0.2 and 1 Pa, respectively.

Considering the diameter of the gaseous molecule *d*_g_ = 0.35 nm (averaged value of air molecules^46^), one can find that (eq. (2)) *l* reaches 100 μm (the typical pore size scale in VIP) at pressure of 10 Pa and 1 cm (the size of the macroscopic opening of the barrier envelope before sealing) at 0.1 Pa, as shown in Fig. 3(b). This indicates that not only the pores in core material cannot be effectively evacuated, even the envelope itself will block the gas flow outward. As a result, even the surface of the core material have pressure larger than the chamber (1 Pa compared to 0.2 Pa at the lowest pressure observable in Fig. 3(a)).

Additional factors cause the increase of the VIP inner pressure in service condition. The inner core pressure of PF core material after sealing is shown in Fig. 3(c) and the venting of the chamber takes place at 45 minutes (only for test purpose) after sealing. Before venting and after sealing, even though the chamber is still at vacuum, the inner pressure dramatically increases to over 2 Pa (the gap at around 1 Pa is due to the switch of different sensor in lower and higher pressure range in the detector). Note that at this stage, the pressure inside the sealed envelope is actually higher than the chamber, so that there is no external pressure on the core material yet. The pressure increase can only be due to the outgassing from the heat sealing of the PE layer. The temperature at the heating band at sealing is measured with a thermocouple detector and peaks at around 170 °C. Even though the temperature is not very high, but even slight outgassing will cause obvious pressure increase at ultralow pressure below 1 Pa. Furthermore, a second pressure increase is observed during the venting of the chamber. This is the result of the volume compression from the atmospheres during venting, at the end of which only the strength of the core material resist the pressure difference between the inner VIP and the atmosphere (Fig. 3(c)). The second increase after venting should be the due to residual gas inside the core material.

The high initial pressure in the VIP can be decreased by getters. A getter equipped with a pressure-penetrable path is used in this work to rapidly decrease the inner pressure after sealing. The core pressure (on the surface of the core material for measurement simplicity) of different core materials are shown in Fig. 3(d). Initial pressure of GF and PF core materials are around 2 Pa, while the pressure of GF-FS core material is around 6 Pa. The high pressure in the fumed silica core material is due to the smaller pore size that further suppress the gas flow during evacuation (larger K_n_). After approximately 2 days of standing time, the pressure in all core materials reach their plateau, showing the efficient adsorption of the getters. Remarkably, when applying 5 getters in the PF VIP with same size, even though the pressure drop faster, the final pressure is almost the same, around 0.3 Pa. In fact, the equilibrium pressure of getters is a function of pressure, typically reaching a plateau at ultralow pressure.^47^ From the results, we inferred that 0.3 Pa is around the plateau equilibrium pressure of the applied getter.

## 4. Aging behavior and dependence on gas permeation

Outgassing or gas permeation has been long blamed for the aging of VIP, while different aging mechanism can be identified in PF VIP. After the pressure is stabilized to the plateau, *K* aging at 70 °C of different types of VIP are measured and shown in Fig. 4(a), in a period of 150 days. The aging of GF and GF-FS VIP coincides well with the reported work: GF VIP has a lower initial conductivity around 1.7 mW mK^−1^, while the aging is much more rapidly (0.03 mW per mK per day) so that it could exceed *K* of GF-FS VIP (increasing rate 0.002 mW per mK per day) after a relative short period. Even though GF-FS VIP also has porosity around 90%,^33^ the fumed silica itself cannot be evacuated due to its close-cell structure.^48–50^ Equivalently, fumed silica can be considered as a solid particle with low bulk *K*, and the core material has a porosity around 50%, which leads to higher initial *K* around 5 mW mK^−1^. For PF VIP, a two-step aging is observed. The aging behavior seem to combine the advantages of GF and GF-FS VIP: a low initial conductivity around 1.8 mW mK^−1^ can be achieved, while the aging is much slower (0.0045 mW per mK per day) than that of GF after a short rise around 0.5 mW mK^−1^ in the first 20 days.

On the other hand, the pressure increase during aging for all core material are almost the same, around 0.1 Pa per day (Fig. 4(b)). The outgassing from the core material is difficult to measure due to the low inner pressure and the soft packaging of VIP. However, it is reasonable to ignore core material outgassing from the pressure increase, as it is unphysical that the outgassing rate from different core material are exactly the same. Considering the same envelope material and size applied for different VIPs, the identical and constant pressure increase rate is attributed to the gas permeation through the barrier envelope. The pressure increase rate due to gas permeation can be expressed by^51^4
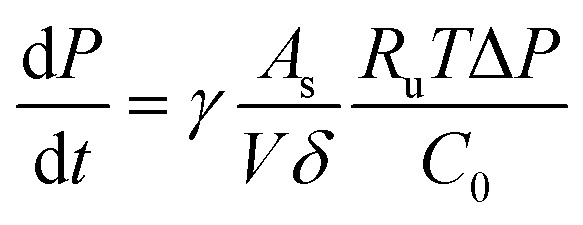
where *γ* is the permeation coefficient, *A*_s_ is the area of permeation surface, *V* is the volume with low pressure and *δ* is the permeation length. *R*_u_ = 8.314 J mol^−1^ K^−1^ is molar gas constant,^52^*C*_0_ = 22.4 L mol^−1^ is the molar volume, *T* = 300 K is the environment temperature and Δ*P* = 10^5^ Pa is the pressure different from the atmosphere. In this way, the pressure increase rate scales with 
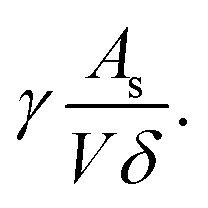


The surface normal and the lateral cross-section of the VIP are discussed separately:

(1) For the surface normal, gas permeates through the whole thickness of the barrier layer. *γ* the two main barrier layers: EVOH and Al are 10^−22^ m^2^ per s per Pa^39,53^ and 10^−27^ m^2^ per s per Pa,^54^ respectively. Using *A*_s_ = 0.15 m^2^, *V* = 0.0015 m^3^, 
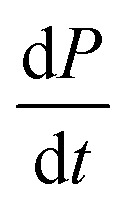
 can be calculated to be 1.37 × 10^−5^ Pa per day for Al layer (7 μm), 0.8 Pa per day for EVOH layer (12 μm) and 0.0025 Pa per day for Al deposited layer (38 nm). As the permeation though the Al layer is much smaller, only EVOH/Al deposited layer accounts for the pressure increase due to permeation. Considering the unavoidable deposition defect (discontinuous layer and dewetting), the permeation rate through the normal surface should be in the range between 0.0025 and 0.8 Pa per day. Note that 
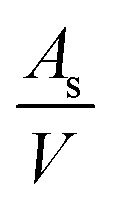
 only depends on the thickness of the VIP.

(2) For the lateral cross-section, the major permeation takes place through the PE layer, which is around *L* = 100 μm, as seen in the inset of Fig. 4(b). Among all layers, PE layer has the thickest thickness and highest *γ* around 10^−17^ m^2^ per s per Pa.^39^ The width of sealing is *δ* = 30 mm, *A*_s_ = 2*La*+2Lb = 0.000156 m^2^. In this way, we have 
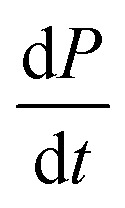
 of 0.033 Pa per day for PE layer. For cross-section, 
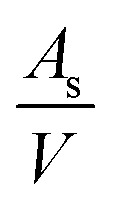
 scales with the ratio of length and thickness of VIP.

It can be found that the permeation through Al layer is much smaller, while PE, Al deposited and EVOH are in a closer range to the experimental value of 0.1 Pa per day. It is reasonable to attribute the pressure increase to only permeation through the barrier envelope.

The pressure increase is much slower under lower temperature and dependent on the permeation rate of polymeric layers. In Fig. 4(c), three VIP made of GF and PF are first kept in room temperature (around 25 °C) and aged under 70 °C at a later time. A pressure increase rate around 0.007 Pa per day can be observed at room temperature and the increase rate at 70 °C is independent of the waiting time in room temperature. Furthermore, when put in back to room temperature (PF B), the increasing rate goes back to the scale in room temperature. The dependence of increase rate on temperature coincides well with the permeation coefficient of PE under different temperature (around 10 times slower from 70 °C to 25 °C (ref. 55)). The pressure increase pattern at different temperature can be predicted following the analysis.

The different aging behavior under same rate of gas permeation comes from the conductivity dependence on increasing inner pressure. To test the dependence relationship in different core materials, microleakages are introduced three VIPs, and the real-time conductivity as the function of inner pressure are measured and shown in Fig. 5(a). The leaking rate is controlled in the scale of 0.1 Pa s^−1^ for simultaneous measurement of thermal conductivity and pressure. Linear relationship can be discovered for all three core material (Fig. 5(a)), with Δ*K*/Δ*P* of 0.133 mW per mK per Pa (GF), 0.074 mW per mK per Pa (PF) and 0.01 mW per mK per Pa (GF-FS), respectively. The linear relationship is tested at low Δ*P* below 1 Pa and still valid (inset in Fig. 5(a) for PF).

The difference in Δ*K*/Δ*P* in VIP corresponds well the constant pressure increase rate and different aging behavior. Depth-coded 3D confocal images of PF and GF based core material are shown in Fig. 5(b) and (c). The average distance in the same plane (same color, *i.e.* depth) can be estimated, which is in the scale of 10–100 μm. It can be confirmed from the images, that GF based core material shows an obviously larger pore size than that of PF one. For GF and PFs, the pore size can be estimated from the overall VIP density and the fiber diameter:5
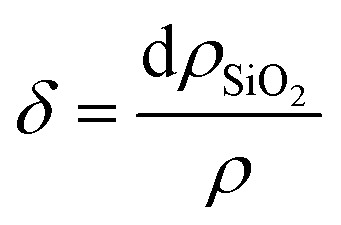
In this way, we have *δ* of 40 μm for PF and 78.3 μm for GF. 
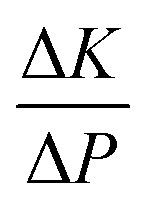
 is expressed by (eqn (3))6
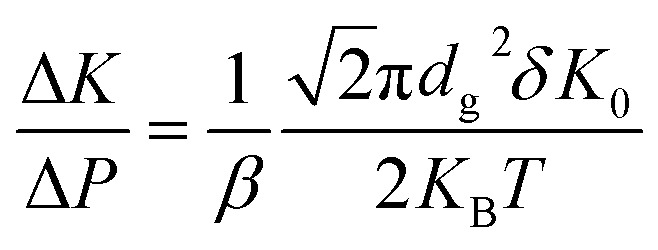


By fitting experimental data in Fig. 5 with eqn (6), using *k*_0_ = 26 mW mK^−1^ of air in standard condition,^56^*β* is around 1.08 for PF and 0.99 for GF, in the same scale as reported value.^25,27^

From the aging measurement (Fig. 4(a)), we have aging rate of 0.002, 0.0045 and 0.03 mW per mK per day for GF-FS, PF and GF VIP, respectively. The experimental results are around 50% of the theoretical value, which are 0.0074 and 0.0133 mW per mK per day for PF and GF, respectively, calculated from the pressure increase rate (0.1 Pa per day). The deviation may come from the different component of the permeated gas, while we use air as the characteristic gas. For our measurement at 70 °C, all VIPs (over 500 VIPs) are storage in an oven set at 70 °C. The relative closed space very likely contains more organic component compared to air. *k*_0_ of organic gas are significantly lower than that of air, in the range of 10–20 mW mK^−1^.^57,58^ This would lead to a smaller *K*_0_, so that smaller Δ*K*/Δ*P*. Applying the experimental value, Δ*K*/Δ*P* is adjusted to be 0.02, 0.045 and 0.3 mW per mK per Pa for GF-FS, PF and GF VIP, respectively.

## 5. Viscoelasticity dominating aging of PF based VIP

**Fig. 6:**
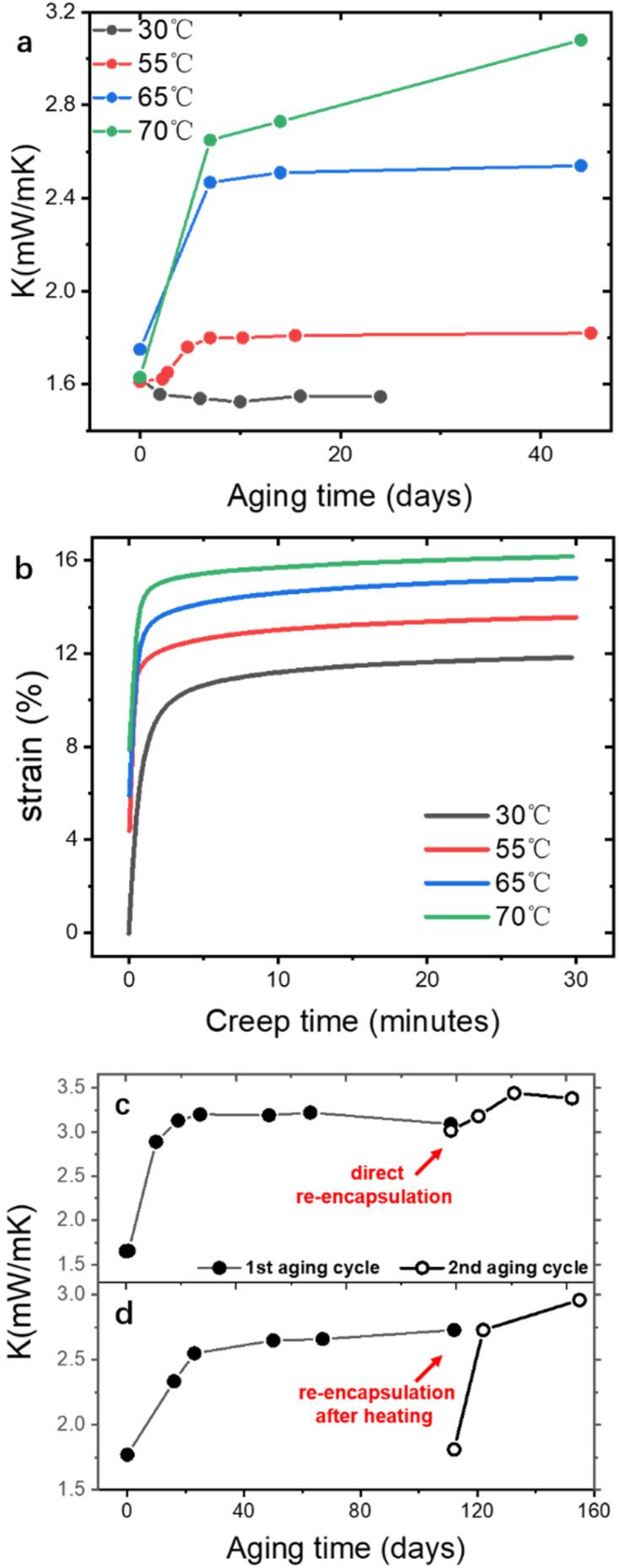
(a) *K* aging at different temperature. (b) and (c): *K* aging in the first cycle and second cycle with (b) direct re-encapsulation and (c) re-encapsulation after heating.

The aging of PF based VIP features two-phase pattern: in the first 20 days, the aging is rather rapid, and then reaches a plateau; after the initial aging period, the aging follows a linear relationship with time, governed by gas permeation. The rapid aging in the initial period is similar to that of the relaxation of viscoelastic polymeric material under fixed load. To exam the role of viscoelasticity in aging, aging tests under different temperature are performed and shown in Fig. 6(a). The clear transition of the two phases take place at around 10 days for aging under 55 to 70 °C. For 30 °C, *K* decreases first in the first 10 days and slowly increases later. Aging at 30 °C is rather slow that we only measured the final result after 200 days (increasing from 1.53 mW mK^−1^ (lowest value at 6 days) to 1.68 mW mK^−1^). Applying the previous conclusion (0.007 Pa per day of pressure increase and Δ*K*/Δ*P* = 0.045 mW per mK per Pa), we estimate the gas conduction contributes 0.06 mW mK^−1^ and remaining 0.09 mW mK^−1^ is contributed by the polymeric fiber itself.

The raw fiber (bundle of single fibers to provide enough force for test) of PF VIP is tested in DMA under creep mode at different temperature, as shown in Fig. 6(b). Plateau strains are reached after different time while the plateau value also depends on the creep temperature. The creep strain γ as the function of creep time t is fitted by Burger's model,^59^ which consisting of Maxwell and Kelvin–Voigt elements:^60^7
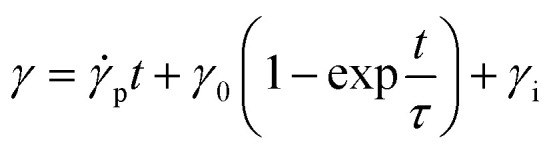
*γ*_0_ and *γ*_i_ are the plateau strain and the instant strain upon loading. *τ* is the characteristic time reaching the plateau strain. *γ̇*_p_ is the creep rate after the plateau, which is related to the dashpot viscosity in Maxwell element. The fitting results are shown in Table 1. Comparing the fitting results, *γ*_m_ is much smaller at 30 °C, while *τ* at 30 °C is almost twice of that at 70 °C. This corresponds well to the results in VIP aging, that smaller plateau *K* and slower creep rate is observed at room temperature. For higher temperature from 55 to 70 °C, *τ* is similar while *γ*_m_ increases with temperature (with almost constant *γ*_0_). This also agrees with aging results, that only plateau value but not time scale changes significantly with increasing temperature over 55 °C. *γ̇*_p_ is rather small and almost constant around 0.1 s^−1^. Considering the gas permeation and different long-term aging rate at different temperatures, the contribution of can be *γ̇*_p_ ignored in VIP aging. Note that for practical storage and application of VIP, the possibly highest temperature is lower than 55 °C. In this way, the actual aging performance of PF based VIP should be more advantageous over PF or GF-FS based VIP.

**Table 1:** The fitting results of the creep curves at different temperatures

*T*	30 °C	55 °C	65 °C	70 °C
*γ*_0_	0.98	0.794	0.795	0.748
*γ*_m_	0.08	0.45	0.61	0.78
*τ*	0.95 min	0.52 min	0.53 min	0.48 min

Remarkably, the insulation property of the aged core materials can be partially recovered by heating. Two VIP after aging of 110 days are: directly re-encapsulated (Fig. 6(c)) and re-encapsulated after heating at 140 °C for 24 hours (Fig. 6(d)), respectively. The initial *K* after direct re-encapsulation is almost same as the aged value (slight drop from 3.4 to 3.3 mW mK^−1^) and the following *K* is around the plateau value around 3.5 mW mK^−1^. This confirms the conclusion from the last section: increasing gas conduction is trivial in short time scale. In our experimental time period (around 100 days), around 0.45 mW mK^−1^ is due to the permeated gas while majority of increasing *K* is from the core material itself. For the re-encapsulated VIP after 90 °C, the initial *K* in the second cycle decreases back to the initial *K* of the cycle (around 2.1 mW mK^−1^). The dramatic drop after heating shows that the majority of the increased *K* is from the structural collapse of the core material, which is heat reversible. However, the second aging (from 110 days to 120 days) is much faster: *K* goes back to over 3 mW mK^−1^ in ten days and then follows the same pattern of the first cycle. The rapidly increased *K* shows that the reversibility of the aged VIP is not complete. We infer that even though the packing of the fiber is recovered, the microscopic structure of the fiber itself has degraded (*e.g.* crystalline structure or orientation^61^).

## 6. Modeling of two-phase aging and long-term performance prediction

Combining solid and gaseous conduction,^62^ and considering the time dependence based on previous discussion (eqn (1), (6) and (7)). The aging behaviors of different materials is modeled by8
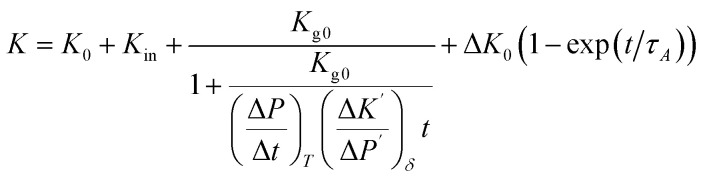


**Fig. 7:**
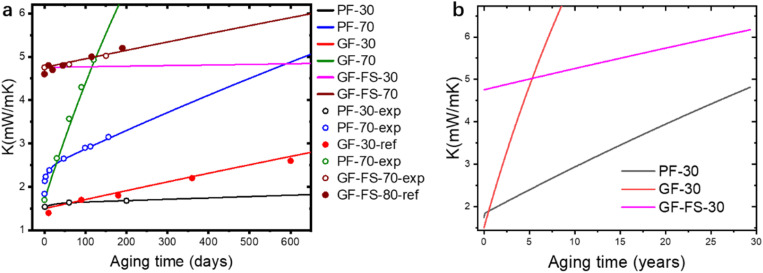
(a) Theoretical (lines) and experimental (empty dots, indicated by -exp) results and reported results from references (solid dots, indicated by -ref) of different VIP at 30 and 70°. (b) Theoretical curves of long-term (30 years) VIP aging at 30°.

*K*_0_ is the experimentally measured value before aging, *K*_in_ is the instant increasing value of *K* (corresponding to *γ*_i_ in fiber creep) and Δ*K*_0_ is the plateau value of the short-term aging. *τ*_A_ is the characteristic time of VIP aging, which should be strongly related to *τ* in fiber creep. 
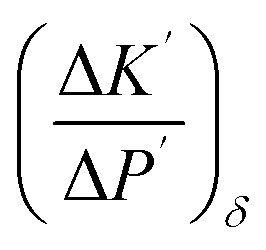
 is core material related coefficient, decided by pore size *δ*. 
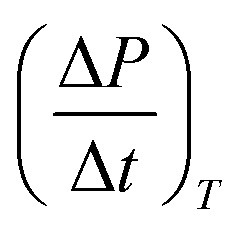
 is the gas permeation rate, decided by temperature *T*. With *P* < 10 Pa (100 days at 70 °C) and *K*_n_ ≫ 1, the second term of the equation becomes linear: 
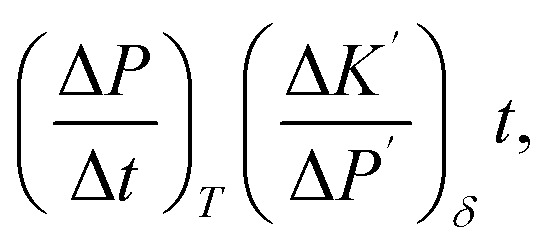
 and the parameters are fitted from the experimental measurements of long-term aging, as shown before. The theoretical and experimental values are shown in Fig. 7(a) (results in 700 days) and (b) (results in 30 years), with parameters presented in Table 2. The parameter (*K*_in_, Δ*K*_0_ and *τ*_A_) of PF-70 is fitted from the aging data in Fig. 4(a) and parameters of PF-30 are estimated based on creep test of fibers. Two sets of aging data from reported work are also included (PF-30-ref. 32 and GF-FS-30-ref. 37) in Fig. 7(a) and also coincides well with the theoretical curve. Note that in both references, the thicknesses of the 2 cm, so that the results are renormalized (eqn (4)) to match our model based on 1 cm VIP (using 
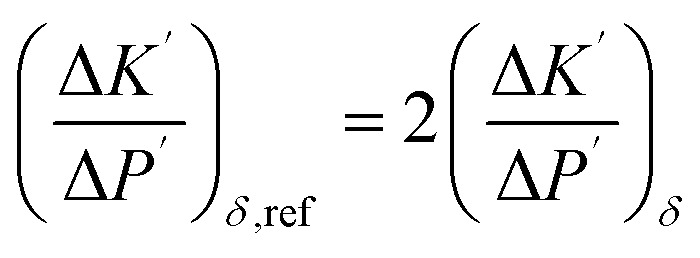
 and same *K*_0_).

**Table 2:** Parameters for aging modeling in Fig. 7

	PF-30	PF-70	GF-30	GF-70	GF-FS-70	GF-FS-70
*K*_0_ (mW mK^−1^)	1.5	1.84	1.5	1.7	4.75	4.75
*K*_in_ (mW mK^−1^)	0.03	0.3	0	0	0	0
Δ*K*_0_ (mW mK^−1^)	0.09	0.33	—	—	—	—
*τ*_A_ (day)	20.8	10.4	0	0	0	0
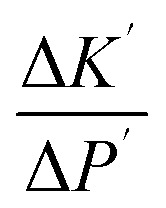 (mW per mK per Pa)	0.045	0.045	0.3	0.3	0.02	0.02
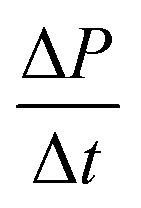 (Pa per day)	0.007	0.1	0.007	0.1	0.007	0.1

It can be found that theoretical model matches well with experimental data. Remarkably, pressure increase under room temperature (common service condition of VIP) is only 0.007 Pa per day, much slower than 70 °C. This corresponds to 0.69, 0.11 and 0.05 mW per mK per year for GF, PF and GF-FS, respectively. In Fig. 7(b), *K* of GF exceeds PF and GF-FS in 50 days and 5 years, respectively. Remarkably, PF increases to only around 5 mW mK^−1^ after 30 years, still lower than GF-FS. From the analysis, PF processes comparable initial *K* to GF (below 2 mW mK^−1^) while comparable aging rate (around 0.1 mW per mK per year) to porous silica under its service condition. The long term aging behavior can be well predicted with the proposed model.

## 7. Conclusion

In this work, the short-term and long-term aging behavior of polymeric fiber (PF) based VIP is studied with *in situ* thermal conductivity *K* and pressure measurements. The contributions to *K* aging are clarified, with glass fiber and glass fiber-fumed silica based VIP as references. PF based VIP shows an unusual two-phase aging behavior: (1) a viscoelasticity dominated short-term aging (in the scale of 10 to 20 days), showing strong temperature dependence; (2) long-term aging governed by gas permeation through the barrier envelope. The long-term aging at room temperature is around 0.1 mW per mK per year, comparable to porous silica-based VIP. Combined with the ultralow initial *K* < 2 mW mK^−1^ and the advantages in healthy/environmentally friendly manufacturing, PF shows great potential as the alternative for traditional VIP core materials.

Technical issues inferred from the experimental observation in this work should be informative for fellow researchers:

(1) Initial high inner pressure in VIP is unavoidable for practical productions (from sealing and insufficient evacuation), and would contributes to a 0.1 mW mK^−1^ for PF VIP and 0.6 mW mK^−1^ for glass fiber VIP. Fortunately, the remaining gases can be effectively absorbed by getters. A balance between getter usage (increasing price) and manufacturing efficiency (longer evacuation time) needs to be judged for future developments.

(2) The aging rate, especially the short-term aging rate of PF VIP, depends strongly on temperature. A low storage temperature before installing of VIP into appliances and building materials is extremely necessary. Furthermore, when practically installed in an insulation layer (constrained with rigid frame), the stress (from the atmosphere) on VIP will be massively shared by the rigid frame. As the creep rate strongly depends on applied load, the aging of PF itself should be even slower than what we show in this work.

(3) Theoretically, one can decrease the fiber diameter (if maintaining same porosity) to decrease pore size, so that slower aging can be achieved without increasing initial *K*. The production and application of thin glass fiber are limited by its health hazards increasing with smaller diameter.^63,64^ Additionally, smaller pore size leads to larger *K*_n_, making it even harder for evacuation. More effective evacuation technology is to be developed to stress this challenge. With further development of evacuation and material processing technologies, PF with smaller diameters will potentially achieve even better aging resistance without increasing health concerning.

## Author contributions

JJ: Original draft, methodology, experiments, data analysis, conceptualization. CL: Review & editing, investigation, conceptualization. QH: Supervision, conceptualization, investigation.

## Conflicts of interest

The authors declare that they have no known competing financial interests or personal relationships that could have appeared to influence the work reported in this paper.

## Data Availability

The data that support the findings of this study are available from the corresponding author upon reasonable request.
